# Preoperative local hemodynamics predict cerebral hyperperfusion syndrome after direct bypass for moyamoya disease: a quantitative CTP study based on ASPECT topography

**DOI:** 10.3389/fneur.2025.1726324

**Published:** 2026-01-15

**Authors:** Jiatong Zhang, Lu Wang, Yi Wang, Yongbo Yang, Zhiyong Shi, Chunhua Hang

**Affiliations:** 1 Department of Neurosurgery, Nanjing Drum Tower Hospital, Affiliated Hospital of Medical School, Nanjing University, Nanjing, Jiangsu Province, China; 2 Neurosurgical Institute, Nanjing University, Nanjing, China; 3 Department of Neurosurgery, Nanjing Drum Tower Hospital Clinical College of Nanjing University of Chinese Medicine, Nanjing, Jiangsu Province, China

**Keywords:** ASPECTS topography, cerebral hyperperfusion syndrome, complications, CT perfusion, EC-IC bypass, moyamoya disease, risk factors

## Abstract

**Background and objective:**

Postoperative cerebral hyperperfusion syndrome (CHS) remains a common and serious complication after extracranial-intracranial (EC-IC) bypass for moyamoya disease (MMD). This study aimed to identify preoperative hemodynamic predictors of CHS using quantitative whole-brain CT perfusion (WB-CTP) analysis.

**Methods:**

The author retrospectively analyzed 103 hemispheres from 89 MMD patients who underwent direct bypass from January 2024 to December 2024. Preoperative WB-CTP scans based on the Alberta Stroke Program Early CT score (ASPECTS) topography were processed to quantify cerebral blood flow (CBF) and time to peak (Tmax) across various brain regions, with the cerebellum serving as the reference. CHS was diagnosed based on clinical and radiological criteria. Univariable and multivariable logistic regression analyses were performed to identify independent predictors, and receiver operating characteristic (ROC) analysis was used to evaluate predictive performance.

**Results:**

Postoperative CHS occurred in 11.7% (12/103) of the included cases. Univariable analysis revealed Suzuki stage, moyamoya vessel density, and Tmax values in the thalamus (THAL) and posterior cerebral artery (PCA) regions as significant factors. Multivariable analysis confirmed advanced Suzuki stage (OR (95% CI), 8.87(1.44–54.45), *p* = 0.018), and lower PCA Tmax (OR (95% CI), 0.03 (0.00–0.69), *p* = 0.029) as independent predictors. ROC analysis demonstrated that combining Suzuki stage and PCA Tmax achieved an AUC of 0.83 (cut-off value = 0.060), indicating good discriminative performance for predicting postoperative CHS.

**Conclusion:**

Advanced Suzuki stage and reduced PCA Tmax are independent risk factors for postoperative CHS after direct bypass in MMD patients. Preoperative ASPECTS-based quantitative CTP analysis can effectively stratify CHS risk and support individualized surgical planning and perioperative management.

## 1 Introduction

Moyamoya disease is a rare, chronic cerebrovascular disease characterized by narrowing or occlusion of the ends of the internal carotid arteries (ICAs) and the formation of a smoky network of abnormal vessels at the base of the brain (1). Extracranial-intracranial (EC-IC) bypass, including direct and indirect bypass, is a recognized effective way to improve cerebral hemodynamics and decrease the risk of subsequent ischemic or hemorrhagic stroke (2, 3). However, cerebral hyperperfusion syndrome (CHS) is a common complication following direct bypass, manifesting with unilateral headache, epilepsy, aphasia, and motor and sensory disorders, which is thought to be associated with transient neurological deficits (TNDs) and even intracranial hemorrhage (4–6). Therefore, exploring the mechanisms and risk factors of postoperative CHS is significant for clinical practice. Previous literature reported the underlying risk factors for postoperative CHS included antegrade hemodynamics source of recipient artery, adult-onset or hemorrhagic-onset, and the mRS score on admission (7). Intraoperative flow monitoring using indocyanine green (ICG) angiography or microvascular ultrasonography can further guide revascularization precision, reducing the risk of postoperative CHS (8, 9). The mechanism underlying postoperative CHS in patients with MMD still remained unclear.

Previous literature reported that whole-brain CT perfusion (WB-CTP), based on Alberta Stroke Program Early CT score (ASPECTS), had been applied to assess hemodynamics automatically in cases with MMD (10). ASPECTS assessment was a semiquantitative scoring system developed for the assessment of acute ischemic changes (EICs) of the blood-supplying of middle cerebral artery (MCA) in stroke patients (11). In this study, we evaluated preoperative local hemodynamics to verify whether it could be used as a predictor of CHS, using WB-CTP quantitative analysis software based on ASPECTS.

## 2 Methods

### 2.1 Patient selection

This study was a single-center retrospective analysis. With the approval of the Ethics Committee of Nanjing Drum Tower Hospital of Nanjing University Medical School, the clinical data of adult MMD patients who underwent superficial temporal artery-middle cerebral artery (STA-MCA) bypass in Nanjing Drum Tower Hospital from January 2024 to December 2024 were retrospectively analyzed. The inclusion criteria were: (1) patients who satisfied the guideline of MMD proposed by the Research Committee on Spontaneous Occlusion of the Circle of Willis of the Ministry of Health, Labor, and Welfare, Japan (12); (2) patients aged more than 18 years old, (3) patients treated by STA-MCA bypass; (4) patients underwent at least 2 CT perfusion (CTP) scans before surgery and 1 week after surgery to evaluate hemodynamics, (5) patients received by postoperative MR angiography (MRA) scan to confirm the anastomotic patency and to exclude ischemia etiology. Exclusion criteria were (1) patients with moyamoya syndrome (MMS) (13), (2) patients with age less than 18 years old, (3) patients with conservative treatment and indirect bypass alone, (4) patients diagnosed with postoperative ischemia related complications, which was diagnosed with diffusion-weighted imaging (DWI) of MRI.

### 2.2 Imaging assessment

#### 2.2.1 WB-CTP imaging protocol

CTP examinations were performed in all MMD patients enrolled. The GE 256-row CT with 80 kV of voltage and 150 mA of current was used, which created 0.625 mm of layer thickness and 256*256 of matrix. The patient was injected with iodine imaging agent intravenously (5 mL/s, a total of 60 mL). After examination, two independent neuroradiologists, who were blinded to each other, analyzed CTP results using Neuro CT aw4.7 software (Siemens Medical System Workstation). The parameters of cerebral blood flow (CBF), and time to maximum (T _max_) were recorded and analyzed.

#### 2.2.2 Data processing and analysis

All perfusion maps and regional quantitative parameters were generated using the Siemens Neuro CT perfusion software (syngo.Via CT Neuro Perfusion, AW 4.7). This platform performs automated brain regional segmentation based on a built-in anatomical atlas.

Perfusion regions were defined using standardized anatomical landmarks with automated atlas-based assignment. The middle cerebral artery (MCA) territory was divided into 10 regions on non-contrast CT according to ASPECTS-based anatomical planes: at the ganglionic level (thalamus and basal ganglia plane), regions included the caudate nucleus (C), lentiform nucleus (L), internal capsule (IC), anterior MCA cortex (M1), insula (I), lateral insular MCA cortex (M2), and posterior MCA cortex (M3); at the supraganglionic level (2 cm above the ganglionic plane), regions included M4, M5, and M6, corresponding to the cortical areas superior to M1–M3, respectively (14, 15). For posterior circulation analysis, the posterior cerebral artery (PCA) territory—primarily encompassing the occipital lobe and posterior temporal–parietal regions supplied by the P2–P3 segments. These regions were defined automatically using the Siemens vascular territory atlas during CTP post-processing. No manual adjustment of regional boundaries was performed.

The data of all these ROIs were calculated and represented as relative values, where cerebellum was selected as reference. The authors artificially defined the frontal lobe as the mean relative value of M1 and M4. The parietal lobe was defined as the mean relative value of M2 and M5. The temporal lobe is defined as the mean relative value of M3 and M6. Mean relative values for the nucleus pulposus, caudate nucleus, and internal capsule were defined as the basal ganglia. Parameters for the insula, medulla oblongata, pons, midbrain, and PCA were also calculated.

#### 2.2.3 DSA assessment in operated hemisphere

Based on the Suzuki Stage (SS) of the ipsilateral hemisphere, patients were classified into early (SS I-II), middle (SS III-IV), and advanced (SS V-VI) stages (16, 17). Additionally, moyamoya vessel density was assessed on DSA and classified into three grades using predefined imaging criteria: None: No visible moyamoya vessels, defined as the absence of basal puff-like collateral networks at the terminal ICA or proximal ACA/MCA. Sparse: Limited moyamoya vessels, defined as a small number of thin, smoke-like collateral vessels confined to the basal cistern or a single vascular territory without forming a confluent network. Dense: Extensive moyamoya vessels, defined as numerous, fine collateral vessels forming a dense, interwoven network radiating in multiple directions from the terminal ICA toward the ACA/MCA/PCA territories. This grading criterion was consistent with our previously published work, in which representative images and detailed definitions were provided (16). These criteria ensured consistent and reproducible categorization across readers (18, 19).

### 2.3 Surgical procedure

All procedures were performed under general anesthesia. A fronto-temporo-parietal craniotomy was utilized to expose the parasylvian cortical arteries (PSCAs) in the frontal, temporal, and parietal lobes, which are common recipient sites for MCA territory bypass. A handheld Doppler probe (MultiDop X, 16 MHz, DWL, Germany) in pulsed-wave mode was used by the surgeon to systematically measure the flow direction and mean systolic velocity (MSV) of each potential recipient artery within the surgical field. The artery with the highest MSV was typically selected as the optimal recipient target. The end to side anastomosis for STA-MCA bypass was completed within 30 min, and patency was subsequently confirmed using intraoperative Flow 800 indocyanine green angiography.

### 2.4 Definition of postoperative CHS

Postoperative CHS was characterized as a set of neurological symptoms following STA-MCA bypass, such as ipsilateral headache, seizures, aphasia, and motor or sensory deficits. The diagnosis of postoperative CHS mainly relied on radiological findings: (1) a marked regional CBF increase around the anastomosis site (with a focal hyperperfusion pattern); (2) clear visualization of the STA-MCA bypass on MRA; and (3) postoperative MRI, including DWI, to rule out ischemia. The MMD case presenting with postoperative CHS included strict blood pressure control (120-130 mmHg), adequate hydration (30–50 mL/kg), prophylactic antiepileptic therapy (100 mg/d), and other supportive measures. Symptoms usually appeared within 2 days after direct revascularization and tended to resolve spontaneously within 2 weeks without permanent deficits.

### 2.5 Statistical analysis

Statistical analysis was performed with SPSS 22.0 (IBM, USA). We used *χ*^2^ tests to examine categorical variables, independent samples t-tests for normally distributed continuous variables, and rank sum tests for ranked or skewed distribution variables. Univariable binary logistic regression analyses were initially performed to screen potential risk factors for postoperative CHS. Variables with a *p* value < 0.05 in univariable analyses were subsequently entered into a multivariable binary logistic regression model using a backward stepwise likelihood ratio method to identify independent predictors of postoperative CHS. ROC analyses were performed to establish cut-off values. A significant level was set at *p* < 0.05.

## 3 Results

### 3.1 Patient characteristics

A total of 89 adult MMD patients (103 hemispheres, patient age range 23–71 years, mean 50 years) (Table 1) met the inclusion criteria for the study. A total 86 (81.13%) patients had ischemic attacks and 20 (18.87%) patients had seizures. There were 45 females (50.56%) and 44 males (49.44%) recruited. In 52 cases, patients were operated in the left hemisphere (50.49%). Patients with infarction history, hypertension and diabetes mellitus included 42, 38 and 12 cases, respectively. The ipsilateral hemispheres in Suzuki stages from early, middle, and advanced stage included 37 (36.0%), 50 (48.5%), and 16 (15.5%) cases. The cases with none, sparse and dense smoky vessels included 17 (16.5%), 38 (36.9%) and 48 (46.6%) cases, respectively. There were no significant differences in the clinical data such as gender, age, clinical type (ischemia/hemorrhage), surgical side, medical history (cerebral infarction, hypertension, diabetes mellitus and moyamoya vessels) density on the operative side between the two groups (*p* > 0.05) (Table 1).

**Table 1:** The basic characteristics of MMD patients recruited.

Variables	Total (*n* = 103)	Non-CHS	CHS	*p*
		Group (*n* = 91)	Group (*n* = 12)	
Age (years)	49.94 ± 9.71	50.32 ± 9.64	47.08 ± 10.13	0.28
Sex, *n* (%)				0.516
Males	52 (50.49)	47 (51.65)	5 (41.67)	
Females	51 (49.51)	44 (48.35)	7 (58.33)	
Onset type, *n* (%)				0.629
Ischemic	85 (82.52)	74 (81.32)	11 (91.67)	
Hemorrhagic	18 (17.48)	17 (18.68)	1 (8.33)	
Stroke, *n* (%)	47 (45.63)	43 (47.25)	4 (33.33)	0.363
Hypertension, *n* (%)	44 (42.72)	38 (41.76)	6 (50.00)	0.587
Diabetes, *n* (%)	13 (12.62)	11 (12.09)	2 (16.67)	1
Surgical side, left, *n* (%)	52 (50.49)	47 (51.65)	5 (41.67)	0.516
Mean mRS on admission	1.16 ± 0.57	1.14 ± 0.57	1.27 ± 0.61	0.499
Suzuki stage, *n* (%)				0.007*
Early stage	37 (36.0)	35 (38.5)	2 (16.7)	
Middle stage	50 (48.5)	46 (50.5)	4 (33.3)	
Advanced stage	16 (15.5)	10 (11.0)	6 (50.0)	
Puff smoke, *n* (%)				0.013*
None	17 (16.50)	17 (18.68)	0 (0.00)	
Sparse	38 (36.90)	29 (31.87)	9 (75.00)	
Dense	48 (46.60)	45 (49.45)	3 (25.00)	

CHS, cerebral hyperperfusion syndrome; mRS, modified Rankin Scale. Bolded values and *: *p* < 0.05.

### 3.2 Clinical outcomes after postoperative CHS

**Figure 1:**
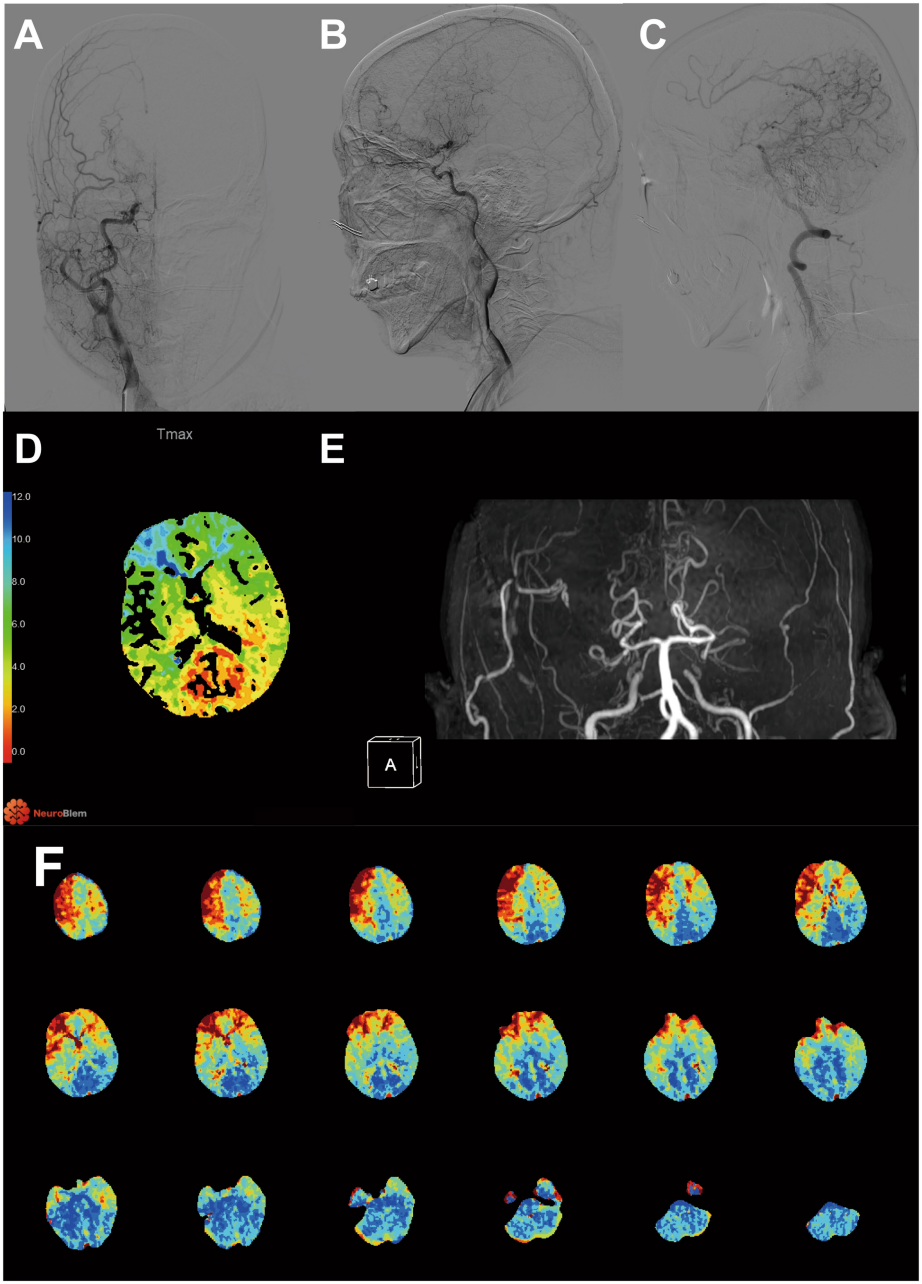
Representative case of CHS: A 56-year-old female patient presented to our hospital with intermittent dizziness and limb weakness over the past 3 years and was diagnosed with MMD. CHS developed 1 day after STA-MCA surgery, presenting as verbal dysfunction. **(A–C)** DSA image before surgery. **(D)** CTP imaging shows a reduction in Tmax values in the PCA region. Red denotes lower Tmax values and blue denotes higher Tmax values. **(E)** MRA demonstrated patency of donor artery. **(F)** Preoperative CTP Tmax map revealed hyperperfused region of right hemisphere. Red denotes higher Tmax values and blue denotes lower Tmax values.

Details of the patients in the CHS group are shown in Table 2. Postoperative CHS occurred in 12 (11.65%) out of 103 surgical hemispheres, including 5 cases of speech dysfunction, 3 cases of aphasia, 1 case of cerebral hemorrhage and 3 cases of severe ipsilateral headache. Figure 1 shows the illustrated cases. CHS episodes occurred between 15 min and 6 days after surgery.

**Table 2:** Detailed information of the patients with cerebral hyperperfusion.

No	Age	Sex	OT	Str	HT	DB	SS	MV	Op side	Symptoms	Time
1	51	F	ISC	0	0	0	5	Sparse	Right	Headache	Po1
2	35	F	ISC	1	1	1	2	Sparse	Right	Verbal dys	Po1
3	60	F	ISC	0	1	0	3	Dense	Right	Verbal dys	Po3
4	56	F	ISC	0	1	0	3	Sparse	Right	Verbal dys	Po1
5	35	M	ISC	0	0	0	2	Sparse	Right	Headache	Po2
6	33	M	ISC	0	0	0	3	Dense	Right	Aphasia	Po2
7	48	M	ISC	1	1	1	3	Dense	Right	ICH, DoC	Po2
8	50	M	ISC	1	1	0	5	Sparse	Left	Unresponsive, verbal dys	Po1
9	59	M	Hem	1	0	0	5	Sparse	Left	Aphasia	Po6
10	57	F	ISC	0	1	0	6	Sparse	Left	Bell’s palsy, verbal dys	Po4
11	45	F	ISC	0	0	0	5	Sparse	Left	Headache, verbal dys	Po6
12	36	F	ISC	0	0	0	5	Sparse	Left	Hemiplegia, aphasia	Po3

OT, Onset type; Str., Stroke; HT, Hypertension; DB, Diabetes; SS, Suzuki stage; MV, moyamoya vessels; Op., operative; ISC, ischemic; Hem., hemorrhagic; dys., dysfunction; Po., postoperative.

### 3.3 Univariable and multivariable analysis of postoperative CHS

**Figure 2:**
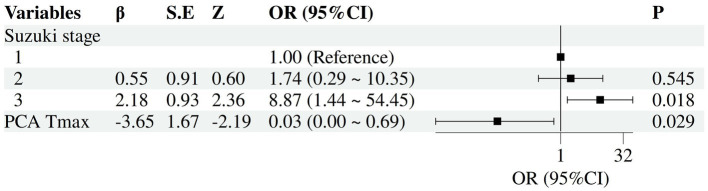
Forest plot of independent risk factors for postoperative CHS. The forest plot illustrates the adjusted odds ratios (OR) and their 95% confidence intervals (CI) for each variable included in the final model.

Univariable and multivariable logistic regression analyses were performed. According to univariable analysis, advanced Suzuki stage, moyamoya vessel density, thalamic Tmax, and PCA Tmax were significantly associated with postoperative CHS (*p* < 0.05) (Tables 1, 3). Variables with *p* < 0.05 were entered into multivariable logistic regression, and Stepwise selection was applied based on the Wald test. During the stepwise procedure, “moyamoya vessel density” had an overall Wald = 2.301, df = 2, *p* = 0.316, which was not significant. Specifically, “sparse” had B = −18.315, SE = 8571.253, Wald = 0, *p* = 0.998, indicating unstable estimation (complete separation). Therefore, this variable was removed. THAL Tmax had B = −0.982, SE = 1.867, Wald = 0.276, *p* = 0.599 in the initial model, not significant, and was also removed. Ultimately, the model retained the variables “Suzuki stage” and “PCA Tmax.” Multivariable analysis showed that advanced Suzuki staging (OR (95% CI), 8.87 (1.44–54.45), *p* = 0.018), and Tmax of PCA (OR (95% CI), 0.03 (0.00–0.69), *p* = 0.029) were statistically significantly associated with the occurrence of CHS (Figure 2).

**Table 3:** Univariable analysis of CTP analysis for postoperative CHS.

Variables	Total (*n* = 103)	Non-CHS (*n* = 91)	CHS (*n* = 12)	*p*
IL CBF	1.21 ± 0.32	1.21 ± 0.33	1.25 ± 0.27	0.67
FL CBF	0.86 ± 0.21	0.86 ± 0.21	0.88 ± 0.20	0.748
PL CBF	0.96 ± 0.23	0.96 ± 0.24	0.93 ± 0.17	0.674
TL CBF	0.83 ± 0.19	0.84 ± 0.20	0.82 ± 0.11	0.627
BG CBF	0.87 ± 0.28	0.87 ± 0.28	0.87 ± 0.21	0.932
MO CBF	0.95 ± 0.27	0.96 ± 0.28	0.89 ± 0.23	0.422
Pons CBF	1.12 ± 0.27	1.12 ± 0.26	1.17 ± 0.36	0.553
MB CBF	1.11 ± 0.31	1.12 ± 0.32	1.05 ± 0.25	0.518
THAL CBF	1.08 ± 0.22	1.08 ± 0.23	1.06 ± 0.20	0.833
PCA CBF	1.65 ± 0.40	1.67 ± 0.40	1.51 ± 0.39	0.191
IL T max	1.27 ± 0.44	1.27 ± 0.44	1.30 ± 0.46	0.804
FL T max	1.41 ± 0.64	1.38 ± 0.63	1.68 ± 0.66	0.121
PL T max	1.41 ± 0.48	1.41 ± 0.49	1.39 ± 0.32	0.892
TL T max	1.21 ± 0.31	1.22 ± 0.31	1.15 ± 0.32	0.463
BG T max	1.04 ± 0.38	1.05 ± 0.38	0.98 ± 0.33	0.508
MO T max	1.03 ± 0.30	1.05 ± 0.30	0.89 ± 0.23	0.084
Pons Tmax	0.82 ± 0.24	0.83 ± 0.24	0.73 ± 0.25	0.165
MB T max	0.90 ± 0.29	0.92 ± 0.30	0.77 ± 0.24	0.096
THAL T max	1.04 ± 0.30	1.07 ± 0.30	0.86 ± 0.25	0.025*
PCA T max	1.11 ± 0.23	1.13 ± 0.23	0.94 ± 0.18	0.006*

IL, insular lobe; FL, frontal lobe; PL, parietal lobe; TL, temporal lobe; BG, basal ganglia; MO, medulla oblongata; MB, mesencephalon; THAL, thalamus; PCA, posterior cerebral artery area. Bolded values and *: *P* < 0.05.

### 3.4 ROC curves and cut-off value of postoperative CHS

**Figure 3:**
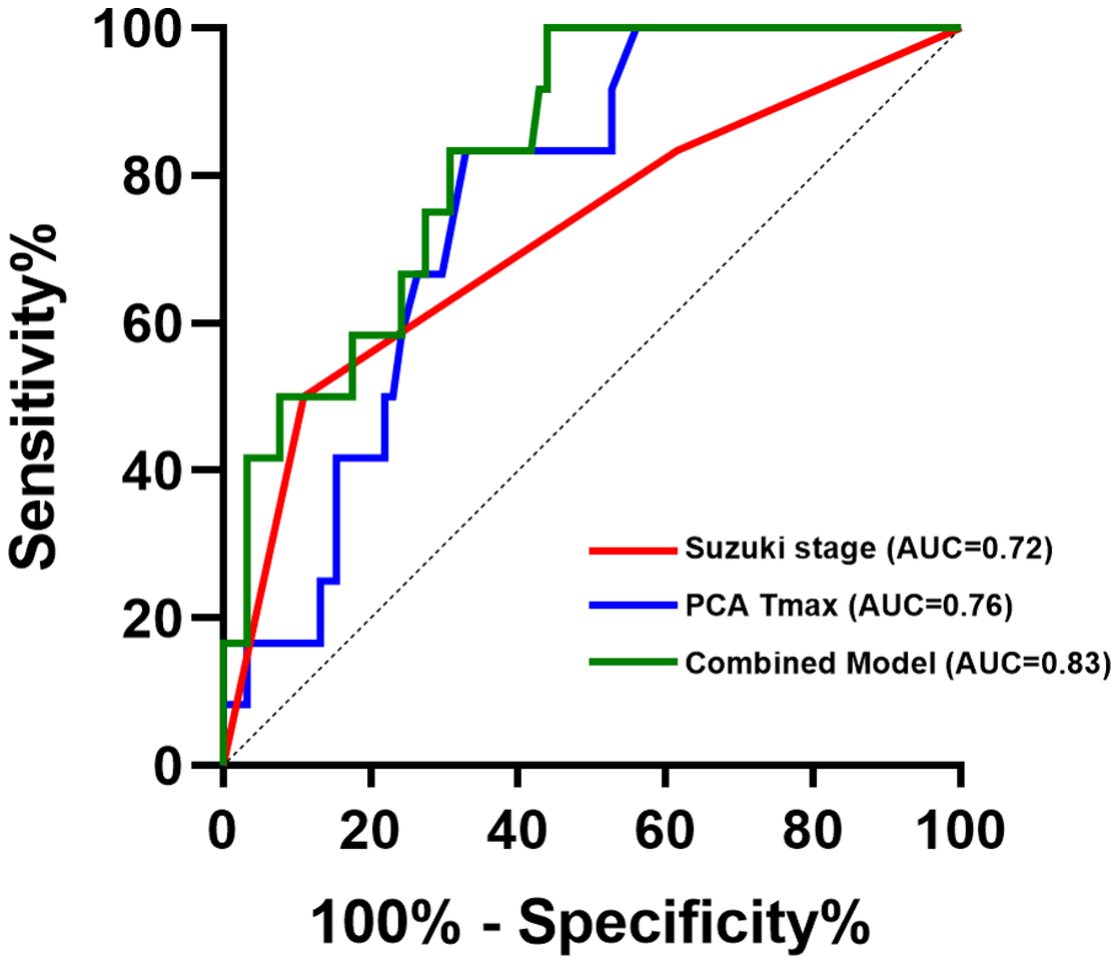
ROC curves of combining Suzuki stage and PCA T_max_.

ROC analysis demonstrated that the multivariable logistic regression model combining Suzuki stage and PCA Tmax achieved an AUC of 0.83 (*p* < 0.000), (cut-off value = 0.060), indicating good discriminative performance for predicting postoperative CHS. In comparison, PCA Tmax alone showed moderate predictive ability, with an AUC of 0.76 (*p* = 0.003) (cut-off value = 1.055). Suzuki stage alone also demonstrated moderate predictive power, yielding an AUC of 0.72 (*p* = 0.015) (cut-off value = 2.5), indicating a significant increase in CHS risk at advanced Suzuki stages (Figure 3).

## 4 Discussion

CHS after STA-MCA bypass is considered to be the most common complication of MMD with a prevalence of 11.3–22.3% (4). In our study, the prevalence of CHS was 11.65%. Recently, increasing attention has been paid to the predictive value of quantitative hemodynamic measurements for CHS. Unlike prior studies focusing on intraoperative hemodynamics (8, 20–22), our quantitative CTP approach provides a preoperative predictive tool, enabling proactive management. Our study found that Tmax parameter of PCA and Suzuki stage were independent predictors of CHS. Combining Suzuki stage and PCA Tmax achieved an AUC of 0.83, indicating good discriminative performance for predicting postoperative CHS. Our findings indicate that a reduced Tmax in the PCA territory, particularly in conjunction with a high Suzuki stage, is a critical independent predictor of CHS. This can be explained by the natural hemodynamic progression of MMD and the physiological impact of STA-MCA bypass.

In early to mid-stage MMD (Suzuki stages I–III), progressive stenosis of the anterior circulation leads to prolonged Tmax in anterior regions due to ischemia, while posterior circulation compensation remains underdeveloped (23, 24). Khan et al. found that as disease advances to Suzuki stages IV–VI, the anterior circulation becomes occluded, moyamoya vessels regress, and the posterior circulation emerges as the primary collateral source (25). During this stage, Tmax in the anterior circulation may exhibit regional variability—prolonging in persistently ischemic areas while improving in zones receiving posterior compensatory flow. Concurrently, Tmax in the posterior circulation shortens significantly, reflecting accelerated flow velocity through high-capacity collateral networks. These networks primarily consist of PCA-ACA anastomoses via the posterior callosal and posterior choroidal arteries, cortical pial-pial collaterals, and thalamic anastomoses originating from the tuberothalamic artery (PcoA) or Percheron artery (PCA) (26–28). The author speculated that the resulting high-flow state in the posterior circulation, while compensatory, introduces hemodynamic vulnerability of the chronically hyperperfused MCA territory, which may already be impaired or maximally vasodilated.

When a direct STA-MCA bypass is performed, the combination of pre-existing high-flow posterior collaterals and new low-resistance STA flow can overwhelm impaired cerebral autoregulation, precipitating cerebral hyperperfusion syndrome (CHS) (29). We further hypothesize that shortened PCA Tmax—indicative of robust posterior-to-anterior shunting—may predict elevated postoperative hemorrhage risk. This risk arises from: (1) microhemorrhage susceptibility at fragile anastomotic junctions (30); (2) increased prevalence of intracranial aneurysms associated with abnormal collateral pathways (31); (3) vessel rupture under combined hemodynamic stress from posterior collaterals and new anterior bypass flow (29). This framework elucidates the mechanistic link between CHS and hemorrhagic transformation in advanced MMD.

Our findings provide a practical, preoperative framework for stratifying CHS risk and personalizing management strategies throughout the perioperative period. Specifically, Patients with lower Suzuki stage may be prioritized for direct bypass procedures (e.g., STA-MCA anastomosis), as their collateral circulation has not yet fully developed and postoperative CHS risk remains manageable. While, the combination of a higher Suzuki stage (V-VI) and a reduced PCA Tmax value identifies a subgroup of patients at substantially increased risk for postoperative CHS. For these high-risk individuals, we recommend a tailored treatment approach. In terms of surgical planning, indirect bypass, such as encephalo-duro-arterio-synangiosis (EDAS), may be preferable, as these techniques promote more gradual hemodynamic adaptation and may mitigate the risk of abrupt hyperperfusion (32).

For patients at high risk of postoperative CHS, strict blood pressure control is required to maintain systolic blood pressure as 120–130 mmHg to avoid blood pressure fluctuations inducing hypoperfusion. However, if the blood pressure is lowered too much, it may lead to contralateral ischaemia in bilateral MMD (9). Enhanced neurological monitoring and timely neuroimaging are also advised. By integrating PCA Tmax metrics with Suzuki staging, clinicians can preemptively tailor interventions across the perioperative continuum, ultimately improving safety and outcomes in high-risk MMD revascularization.

Recently, the mechanism proposed by Jung et al. may complement the findings of our study (33). Jung et al. emphasized reduced cerebral vascular reserve in the temporal cortex, indicating that this region frequently remains in a state of chronic maximal vasodilation with significantly impaired autonomic regulation. Combined with our findings, a more comprehensive hypothesis may be proposed: in advanced MMD, the temporal cortex becomes a low-reserve, high-susceptibility recipient region, while the posterior circulation evolves into a high-perfusion source through robust collateral pathways. When a direct anterior temporal artery-middle cerebral artery bypass is established, the pre-existing high perfusion state of the posterior circulation combines with the newly introduced low-resistance bypass flow. This combination may exceed the limited vasoregulatory capacity of the temporal cortex, inducing regional perfusion overload and blood–brain barrier disruption, ultimately leading to cerebral hyperperfusion syndrome. This complementary framework suggests that concurrent assessment of regional vascular reactivity and spatially resolved perfusion dynamics indicators, such as Tmax, representing hemodynamic load, enables more precise identification of patients at the intersection of “high perfusion” and “low tolerance.” This cohort exhibits the highest postoperative risk for CHS.

### 4.1 Limitations

This study represents the first application of quantitative CT perfusion parameters for region-specific prediction of postoperative CHS. However, several limitations should be considered. First, the findings are derived from a single-center cohort and may be influenced by selection bias. Second, the retrospective design inherently limits causal inference. Third, although the overall cohort included hundreds of cases, the number of observed CHS events remained low due to the condition’s relatively rare incidence. A larger sample size with more CHS cases may yield more robust and clinically significant predictors in the future. Although analyses were performed at the hemispheric level (103 hemispheres from 89 patients), some patients contributed bilateral data, potentially violating the assumption of independence. While clustering-adjusted methods (e.g., mixed-effects models or generalized estimating equations) would be preferable, the small number of CHS events led to model instability and unreliable estimates. Therefore, hemispheres were analyzed as independent units, given the known asymmetry of hemispheric hemodynamics and surgical outcomes in moyamoya disease. This limitation should be addressed in future studies with larger sample sizes. Finally, the assessment of posterior circulation compensation in the present study was Semi-quantitative in nature and based primarily on relative values of CTP parameters, which may limit the precision of evaluating its contribution to postoperative hyperperfusion. Future studies incorporating quantitative, DSA-based hemodynamic parameters, such as flow velocity, flow volume, and standardized collateral grading, will be essential to more accurately characterize posterior circulation compensation and to further elucidate its role in postoperative hyperperfusion.

## 5 Conclusion

Advanced Suzuki stage and Tmax value of PCA are potential risk factors for postoperative CHS. Quantitative preoperative CTP analysis is helpful in assessing hemodynamics and predicting CHS. our study provides data and perspectives for predicting postoperative CHS and selection of treatment modalities in patients with MMD.

## Data Availability

The original contributions presented in the study are included in the article/supplementary material, further inquiries can be directed to the corresponding authors.

## Glossary

ASPECTS: Alberta Stroke Program Early CT score
CBF: Cerebral blood flow
CHS: Cerebral hyperperfusion syndrome
CI: Confidence interval
CTP: CT perfusion
DSA: Digital subtraction angiography
EC-IC: Extracranial-intracranial
MCA: Middle cerebral artery
MMD: Moyamoya disease
MRA: MR angiography
MTT: Mean transit time
OR: Odds ratio
PCA: Posterior cerebral artery
CBF: Relative cerebral blood flow
ROI: Region of interest
ROC: Receiver operating characteristic
Tmax: Relative time to peak
SD: Standard deviation
THAL: Thalamus
TNDs: Transient neurological deficits
WB-CTP: Whole-brain CT perfusion
